# Treatment with inhaled antibiotics in bronchiectasis, side effects, and evaluation of the tolerance test; analysis from the BATTLE randomized controlled trial

**DOI:** 10.1111/crj.13663

**Published:** 2023-07-17

**Authors:** Lotte C. Terpstra, Daphne van der Geest, Inez Bronsveld, Harry Heijerman, Wim G. Boersma

**Affiliations:** ^1^ Department of pulmonary medicine Northwest Clinics Alkmaar Alkmaar The Netherlands; ^2^ Department of pulmonary medicine University Medical Centre Utrecht Utrecht The Netherlands

**Keywords:** airway hyperresponsiveness, bronchiectasis, side effects, tobramycin inhalation solution, tolerance test

## Abstract

**Introduction:**

Tobramycin inhalation solution (TIS) is a treatment option for patients with frequent exacerbations of bronchiectasis. A possible side effect of TIS is the development of chronic cough and bronchospasm, whereby the guidelines suggest a (in hospital) tolerance test with the first dose of TIS. However, data on respiratory adverse events are not consistent. In the present analysis from the BATTLE study (NCT02657473), we evaluated the added value of the tolerance test and aimed to observe the development of inhaled treatment related bronchial hyperreactivity.

**Methods:**

Fifty‐seven patients from the BATTLE study were analyzed. Patients were randomized to receive TIS or placebo OD for 1 year. A tolerance test was performed with spirometry measurements before and after the first dose and with a bronchodilator in advance. Adverse events were strictly monitored.

**Results:**

Fifty‐seven patients (100%) passed the tolerance test with no decrease in spirometry measurements or development of local intolerability. During the study treatment, a total of five TIS‐treated patients (17.8%) withdrew due to airway hyperresponsiveness after a mean of 9.2 (SD13.9) weeks and one placebo‐treated patient (3.5%) after 2 weeks (TIS vs. placebo; *p* = 0.66). The other TIS‐related adverse events were not clinically significant.

**Conclusion:**

The use of inhaled medication is well tolerated in the heterogenous bronchiectasis population, without signs of airway hyperresponsiveness after the first dose of inhaled medication. From this observation, it can be concluded that there is no additional value for this advised tolerance test. However, closely monitoring on adverse effects during the first weeks after starting TIS is recommended.

## Graphical abstract

In this sub‐analysis from the BATTLE RCT, the use of inhaled medication (TIS or NaCl 0.9%) was evaluated whereby no signs of airway hyperresponsiveness were observed after the first dose. It can be concluded that there is no additional value for this advised tolerance test. However, during study treatment, airway hyperresponsiveness was seen after a mean of 9.2 (SD 13.9) weeks, whereby closely monitoring on adverse/site effects during the first weeks of TIS is recommended.

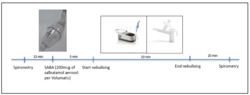

## 1 INTRODUCTION

Bronchiectasis is characterized by the presence of dilated bronchi and chronic inflammation, which leads to persistent coughing and sputum production with recurrent exacerbations.^1^ Long‐term inhaled antibiotics are recommended for the frequent exacerbating bronchiectasis patients, with additional evidence for treatment with tobramycin inhalation solution (TIS).^1^, ^2^, ^3^ The inhaled administration of antibiotics can provide a consistent deposition of high antibiotic concentrations directly to the side of infection, with a lower risk of toxicity or systemic adverse events.^2^, ^4^ However, possible side effects of the use of inhaled TIS are chronic cough and bronchospasm, which have been reported in up to 70% of patients treated with TIS in previous studies.^5^, ^6^, ^7^ However, data on the respiratory adverse events were not consistent, and in contrast to these studies, two recent conducted randomized controlled trials (RCTs) observed no significant bronchial hyperreactivity after the use of TIS.^3^, ^8^ In addition, no significant increase in the development of bronchospasms was found in a recent published meta‐analysis, whereby RCTs were included with TIS as well as other variants of inhaled antibiotics.^2^ Because of the lack of consistent data, our current available guidelines suggest a clinical (in hospital) tolerance test with the first dose of the inhaled antibiotics to observe the possible occurrence of bronchospasm.^1^, ^9^ This observation often includes spirometry measurements before and after a supervised test dose of inhaled antibiotics. Furthermore, the inhalation of a short‐acting bronchodilator before the use of inhaled antibiotics is advised to prevent bronchospasm. However, it remains unclear whether conducting this tolerance test with the first dose of inhaled antibiotics can prevent patients from experiencing persistent cough and/or airway obstruction directly or later during the inhaled treatment. In the present analysis from the BATTLE study,^10^ we evaluated the added value of the suggested (in hospital) tolerance test and aimed to observe and analyze the development of inhaled treatment‐related bronchial hyperreactivity and other side effects.

## 2 METHODS

### 2.1 Study population

In the present analysis, data from the BATTLE RCT were included.^10^, ^11^ The BATTLE RCT (clinical trials.gov number: NCT02657473) was conducted in the Netherlands between 2016 and 2019.^10^, ^11^ A total of 58 bronchiectasis patients with frequent exacerbations were included and randomized to receive TIS (300 mg/5 mL) once daily (OD) or placebo OD (5 mL saline, 0.9%) for 1 year by using the InnoSpire Deluxe compressor (Philips Respironics) with a SideSteam Plus nebulizer with filter and mouthpiece, which is generally used in the Netherlands for inhaled antibiotics.^12^ The study protocol and the results of the BATTLE study were previously published.^10^, ^11^

### 2.2 Objectives

The primary objective of this study is to evaluate the presence of airway hyperresponsiveness during the tolerance test in patients treated with TIS as compared with placebo. Secondary objectives were time to first signs of airway hyperresponsiveness for patients treated with TIS as compared with placebo, number of treatment‐related adverse events, and etiology of bronchiectasis patients in whom intolerability of the inhaled medication occurs.

### 2.3 Tolerance test

Patients were clinically stable at the start of the study, and a tolerance test with the first dose of the study medication was performed for each patient at the outpatient ward. Patients were excluded if they failed the tolerance test. Study visits were planned every 3 months for 1 year, and a diary card was used every week to obtain information on the development of possible side effects (Figure S1).

All patients underwent this supervised test dose by using the InnoSpire Deluxe at the outpatient ward at visit 0 (the start of the study) to assess the occurrence of local intolerability. Patients continued their own maintenance inhaled medication during the tolerance test and next to the study medication.

A spirometry measurement was performed 20 min before the first dose of the study medication. All patients received a short‐acting beta agonist (200 mcg salbutamol dose aerosol with aerochamber) 5 min before the study medication. Spirometry measurements were repeated 20 min after the use of the inhaled study medication (Figure S2). Spirometry measurements were expressed in forced vital capacity (FVC) and forced expiratory volume in one second (FEV1) in liters and percent of predicted. Study discontinuation by airway hyperresponsiveness was defined as: (1) a decrease in FEV_1_% of the predicted 20% following the study medication; (2) and/or SO2 <90% at the fingertip; (3) and/or signs of bronchospasm. Signs of bronchospasm were defined as cough, dyspnea, wheezing, and signs of airway obstruction by clinical examination. During the study period, all patients used the short‐acting beta agonist (200 mcg salbutamol dose aerosol with aerochamber) daily before the study medication.

### 2.4 Statistical analysis

Statistical analysis was conducted using IBM SPSS 25 for Windows. Discrete variables were presented as counts (percentage) and continuous variables as mean with ±standard deviation (SD) if normally distributed or median with interquartile range (IQR) if not normally distributed. Comparison between groups was performed using the independent samples test if normally distributed and the Mann–Whitney U test if not normally distributed. For comparison between groups with multiple variables, a chi‐square test was used. Within each treatment group, changes in FEV1 and FVC pre and post study medication were analyzed using the Wilcoxon signed rank test. A *p*‐value *p* < 0.05 was considered statistically significant.

## 3 RESULTS

### 3.1 Patients

Fifty‐seven patients out of 58 who participated in the BATTLE study^10^ were included in this analysis. One patient (1.7%) was excluded during the first visit of the study due to other medical problems. An overview of the patient characteristics is shown in Table 1. Baseline patient characteristics were well balanced, except for maintenance (stable) doses of prednisolone <10 mg, which were more often used in the TIS group (*p* = 0.02). The mean age of the study population was 68.6 years (SD 7.2) for patients treated with TIS and 65.8 years (SD14.3) for placebo‐treated patients. No differences were found in smoking status, the etiology of bronchiectasis, and the use of inhaled medication (Table 1).

**TABLE 1:** Patient characteristics.

	Tobramycin	Placebo
	*n* = 28	*n* = 29
Age, mean (SD), year	68.6 (7.2)	65.8 (14.3)
Female, *n* (%)	14 (50.0)	19 (65.5)
Smoking status, *n* (%)		
Current	2 (7.1)	1 (3.4)
Former	14 (50.0)	16 (55.2)
Never	12 (42.9)	12 (41.4)
Etiology, *n* (%)		
Post‐infective	5 (17.9)	8 (27.5)
Idiopathic	6 (21.4)	4 (13.8)
COPD	6 (21.4)	6 (20.7)
Asthma	3 (10.7)	5 (17.2)
Immunodeficiency	4 (14.3)	3 (10.3)
Rheumatic disease	1 (3.6)	1 (3.4)
Primary ciliary dyskinesia	0	1 (3.4)
Alpha‐1 antitrypsin deficiency	0	1 (3.4)
Yellow nail syndrome	1 (3.6)	0
Aspiration	2 (7.1)	0
Charlson comorbidity index, *n* (%)		
1 or 2	20 (71.4)	23 (79.3)
3 or 4	8 (28.6)	6 (20.6)
Maintenance AZM during the study, *n* (%)	8 (28.6)	4 (13.8)
Maintenance prednisolone during the study <10 mg, *n* (%)	6 (21.4)	0
Maintenance immunoglobulin therapy, *n* (%)	1 (3.4)	1 (3.4)
Inhaled medication, *n* (%)		
SABA	17 (60.7)	20 (69.0)
SAMA	3 (10.7)	9 (31.0)
LABA	19 (67.9)	19 (65.5)
LAMA	10 (35.7)	12 (41.4)
ICS	19 (67.9)	21 (72.4)
No use of SABA before tolerance test	1 (3.4)	1 (3.6)

*Note*: Data are presented as *n* (%), mean (SD), or median (IQR).

Abbreviations: AZM, azithromycin; ICS, inhalation corticosteroids; IQR, interquartile range; LABA, long acting β agonist; LAMA, long‐acting anticholinergics; SABA, short acting β agonist; SAMA, short acting anticholinergics; SD, standard deviation.

### 3.2 Tolerance test

The tolerance test was performed in 57 patients, whereof 2 (3.5%) patients did not receive salbutamol dose aerosol in advance. All patients passed the tolerance test without severe airway obstruction (defined as a decrease in FEV_1_% predicted of >20% and/or SO2 <90%, measured at the fingertip) after the first dose of the study medication. In the total population, a minimal improvement of FEV1% of predicted from 68.3% (SD 23.9) to 69.1% (SD 23.3) was found after the tolerance test, with no differences in FVC (before and after: 86% of predicted [SD 19.0]). One (3.5%) TIS‐treated patient mentioned a bad taste after inhalation. No other treatment‐related adverse events were mentioned or observed during or directly after the tolerance test. An overview of the spirometry measurements during the tolerance test for the TIS‐ and placebo‐treated patients is shown in Table 2. No significant differences were found before and after the use of the inhaled medication between both treatment groups (*p* = 0.62 for FEV_1_% of predicted and *p* = 0.23 for FVC% of predicted).

**TABLE 2:** Tolerance test.

	Tobramycin			Placebo		
	Before	After	*p*‐value	Before	After	*p*‐value
FEV1 liters	1.8 (0.7)	1.8 (0.7)	0.28	1.9 (0.7)	1.9 (0.7)	0.45
FEV1% of predicted	65.1 (24.2)	67.0 (23.4)	0.18	71.4 (23.7)	71.3 (23.5)	0.58
FVC liters	2.9 (0.8)	3.0 (0.8)	0.17	3.1 (0.9)	3.1 (0.9)	0.61
FVC% of predicted	84.4 (19.4)	86.7 (19.8)	0.11	89.4 (18.3)	87 (24.6)	0.46

*Note*: Spirometry measurements before and after the inhaled study medication. Tobramycin (*n* = 28), placebo (*n* = 29). Data are presented as mean (SD).

Abbreviations: FEV1, forced expiratory volume in one second; FVC, forced vital capacity; SD, standard deviation.

A total of 16 patients (28%) were known to have FEV1% of predicted <50% before the use of the inhaled study medication, of which 11 patients (68.8%) were treated with TIS. For this subpopulation with a FEV1 <50% of predicted, a significant improvement was found in the FEV1% of predicted from 39.1% (SD 7.4) to 41.5% (SD 8.9) during the tolerance test (*p* = 0.01) (not shown). Only one TIS‐treated patient has been withdrawn after 3 weeks due to airway hyperresponsiveness. No spirometry measurement was performed at the time of the outage.

### 3.3 Reasons for study discontinuation

During the study treatment, a total of 18 (31.6%) patients withdrew from the study: 11 (19.3%) TIS‐treated patients after a mean of 10 weeks (SD 10.6) and 7 (12%) placebo‐treated patients after a mean of 7.7 (SD 6.4) weeks (*p* = 0.22). An overview of the side effects is shown in Table 3. A total of five TIS‐treated patients (17.8%) were withdrawn from the study due to airway hyperresponsiveness defined as dyspnea and/or signs of airway obstruction and/or severe cough after a mean of 9.2 (SD 13.9) weeks. One placebo‐treated patient (3.5%) experienced signs of airway obstruction during treatment and withdrawn from the study after 2 weeks. No spirometry measurements were performed at the time of withdrawal. Though spirometry measurements during the tolerance test for these five TIS‐treated patients and one placebo‐treated patient improved, there were no other signs of airway hyperresponsiveness.

**TABLE 3:** Overview of reasons for study discontinuation.

	Tobramycin (*n* = 28)	Placebo (*n* = 29)	*p*‐value
Withdrawn from the study (*n*, %)	11 (39)	7 (24)	0.22
Number of weeks (mean, SD)	10 (10.6)	7.7 (6.4)	0.62
Range	1–34 weeks	1–18 weeks	
Reasons for study discontinuation			
Airway obstruction/dyspnea	3 (27)	1 (14)	ns
Severe cough	2 (18)	0	
Allergic reaction	1 (9)	0	
Bad taste after inhalation	1 (9)	0	
Renal impairment	1 (9)	0	
Tinnitus	0	1 (14)	
Headache after inhalation	0	1 (14)	
No effect	2 (18)	3 (43)	
Intensive study schedule	1 (9)	1 (14)	

*Note*: Data are presented as *n* (%) or mean (SD).

Abbreviations: ns, non‐significant; SD, standard deviation.

Of these six patients who stopped the study, one TIS‐treated patient (16.7%) was an actual smoker, whereas the other patients never smoked. For one TIS‐treated patient, the etiology of bronchiectasis was defined as asthma; two patients were known to have idiopathic bronchiectasis, one post‐infective, and one with immunodeficiency‐related bronchiectasis. The placebo‐treated patient was known to have asthma‐related bronchiectasis. None of them was known to have chronic obstructive pulmonary disease (COPD) (Table S1).

Regarding specific adverse events based on the aminoglycoside safety profile in the patients who have been withdrawn, no TIS‐treated patients experienced ototoxicity; one patient (9%) showed reversible renal impairment after a treatment period of 9 months; and one patient (9%) showed an allergic reaction with swelling and irritation of the lips after the use of TIS, which was not seen during the tolerance test. One (9%) placebo‐treated patient experienced ototoxicity with tinnitus during the treatment period. For this patient, an audiogram was performed, which showed no signs of medication related ototoxicity. An overview of all the adverse events and serious adverse events in the total population is shown in Table S2.

## 4 DISCUSSION

The present sub‐analysis of the BATTLE study^10^ showed that the use of inhaled medication (TIS or NaCl 0.9%) is well tolerated in the heterogenous bronchiectasis population, without signs of airway hyperresponsiveness after the first dose of inhaled medication.

A total of 57 patients underwent a tolerance test with the first dose of the inhaled medication (TIS or placebo) with salbutamol DA in advance. None of these patients showed a lung function decline or other signs of airway hyperresponsiveness after the tolerance test. Despite a normal tolerance test, airway hyperresponsiveness developed especially during the first weeks of maintenance treatment, whereby in our study, six (10.5%) out of the 57 patients showed airway obstruction, dyspnea, or chronic cough. None of the other adverse effects of TIS during the study could be predicted by the tolerance test.

Based on this observation, no additional value was seen for this advised tolerance test; however, closely monitoring in the first weeks after the start of maintenance inhalation treatment seems more relevant.

Higher percentages, up to 30%, in the occurrence of airway hyperresponsiveness were described in previous studies with TIS; however, bronchodilation in advance was not standard used in these studies.^5^, ^6^

Only in a few studies in bronchiectasis was a tolerance test or supervised test dose performed before the use of an inhaled treatment option.^13^, ^14^ After the first dose of inhaled mannitol, a decline in lung function, oxygen desaturation, or use of bronchodilator was described up to 16.5% and was reported as screen failure.^13^ In a RCT with colistin, no lung function decline was found after the first dose of inhaled colistin, though only one placebo‐treated patient showed a decrease in FEV1 >15%. In line with the results of our study, airway hyperresponsiveness developed in the first weeks after the start of inhaled colistin in five (7%) patients.^14^

Maintenance use with inhaled antibiotics is time‐consuming and takes about 20–30 min a day, and it includes the preparation of the device, the use of the inhaled medication, and the cleaning protocol afterwards. This time‐consuming therapy has been a reason for a total of seven patients (38.9%) to be withdrawn from our study. They experienced an insufficient effect of the treatment in combination with a too intensive study schedule. Other adverse events obtained in our study were (reversible) renal impairment after the use of TIS, which was at comparable rates to those described in the recently published iBEST study^8^ in patients treated with the OD dosing schedule; the twice daily (BID) dosing schedule showed increased rates of renal impairment.^8^ In our study, ototoxicity, which led to study discontinuation, was found in one patient (9%) and was not TIS treatment‐related. Two patients (7%, not shown) in the total TIS population mentioned tinnitus, but this was mild and transient and did not result in a change in study medication. Previous studies showed higher rates of ototoxicity, which is probably related to the twice daily dosing schedule and therefore dose‐dependent.^7^, ^8^, ^15^ However, in our study, only patients who mentioned hearing complaints underwent audiometry; therefore, our results must be interpreted with caution.

In our study, bronchiectasis patients with pre‐existing low spirometry measurements were included (FEV1 < 50% of predicted), and even in this population, the use of the inhaled study medication was well tolerated. Only one patient dropped out of the study after a period of 3 weeks due to airway hyperresponsiveness despite the use of a bronchodilator.

In conclusion, the use of inhaled medication (TIS or NaCl 0.9%) is well tolerated in the heterogenous bronchiectasis population without signs of airway hyperresponsiveness after the first dose of inhaled medication. From this observation, it can be concluded that there is no additional value for this advised tolerance test. However, closely monitoring adverse/site effects during the first weeks of TIS is recommended.

## AUTHOR CONTRIBUTIONS

W. G. Boersma and Lotte C. Terpstra designed and drafted the manuscript. All authors were involved in revising the manuscript and have given final approval of the version to be published.

## CONFLICT OF INTEREST STATEMENT

WGB reported grants paid to his institution from GlaxoSmithKline and reported consulting fee for the Adviesraad 2021. IB and HH reported grants form Longfonds and CF fonds paid to their institution. LCT and DG have nothing to disclose.

## ETHICS STATEMENT

This study is a sub analysis of the BATTLE study. The BATTLE study protocol was reviewed and approved by independent ethics committees and institutional review boards from all the six participating centres. The study was performed in accordance with the Good Clinical Practice guidelines, the International Conference on Harmonization guidelines and the most recent version of the Declaration of Helsinki. Written informed consent for participation and publication of our results was obtained from all the participants at the screenings visit. The BATTLE study is registered on Clinical trials.gov number: NCT02 657473.

## Supporting information


**Figure S1.** Study schedule. Abbreviations: Tobramycin inhalation solution (TIS); Once daily (OD); Lower respiratory tract infections – Visual Analogue Scale (LRTI‐VAS); Leicester cough questionnaire (Leicester cough); Quality of life bronchiectasis questionnaire (QoL‐B).
**Figure S2.** Time schedule of the tolerance test. Abbreviations: SABA: short‐acting beta agonist.
**Table S1.** Analysis of the sub population who withdrawn from the study due to airway hyperresponsiveness.
**Table S2.** Overview of the adverse events and serious adverse events in the total population

## Data Availability

The data that support the findings of this study are available on request from the corresponding author. The data are not publicly available due to privacy or ethical restrictions.
