# Supplementary material for: Treatment with inhaled antibiotics in bronchiectasis, side effects, and evaluation of the tolerance test; analysis from the BATTLE randomized controlled trial
Source: Clin Respir J. 2023 Jul 17;17(8):748–53. doi: 10.1111/crj.13663 (PMC10435932; doi:10.1111/crj.13663)
Supplement: Supplementary file 1 — Figure S1. Study schedule. Abbreviations: Tobramycin inhalation solution (TIS); Once daily (OD); Lower respiratory tract infections – Visual Analogue Scale (LRTI‐VAS); Leicester cough questionnaire (Leicester cough); Quality of life bronchiectasis questionnaire (QoL‐B). Figure S2. Time schedule of the tolerance test. Abbreviations: SABA: short‐acting beta agonist. Table S1. Analysis of the sub population who withdrawn from the study due to airway hyperresponsiveness. Table S2. Overview of the adverse events and serious adverse events in the total population [file CRJ-17-748-s001.docx]

**SUPPLEMENTAL MATERIAL**

**
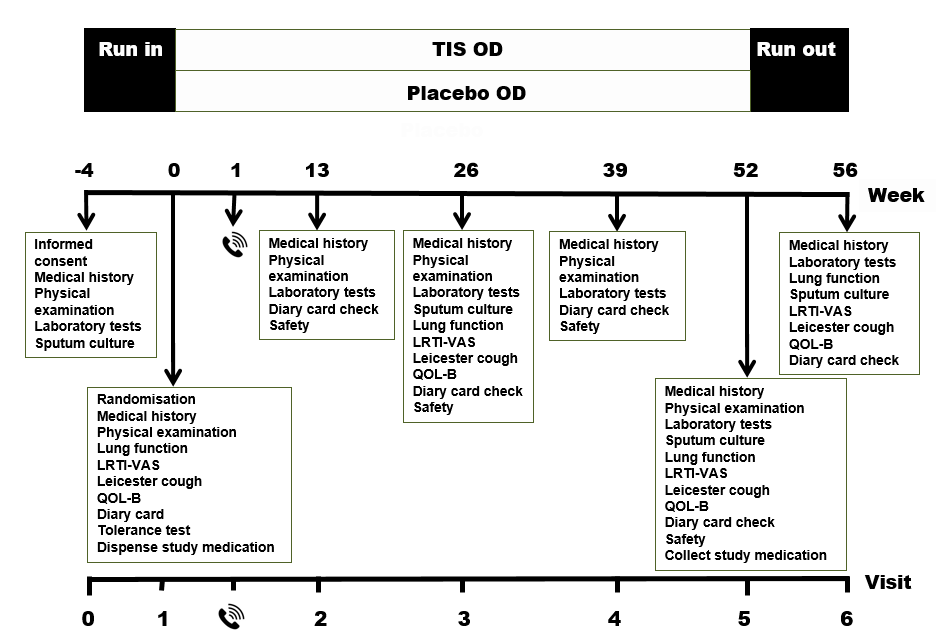
Supplemental 1. Study schedule**

Figure 1. Study schedule. Abbreviations: Tobramycin inhalation solution (TIS); Once daily (OD); Lower respiratory tract infections – Visual Analogue Scale (LRTI-VAS); Leicester cough questionnaire (Leicester cough); Quality of life bronchiectasis questionnaire (QoL-B).

**
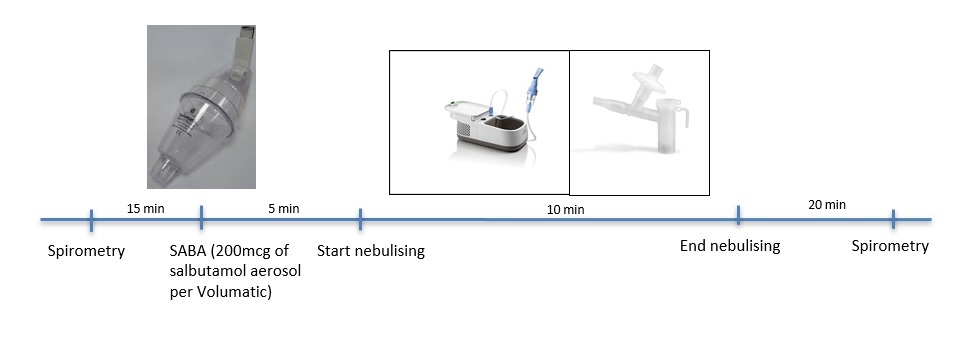
Supplemental 2. Time schedule of the tolerance test**

Figure 2. Time schedule of the tolerance test. Abbreviations: SABA: short-acting beta agonist.

**Supplemental 3. Analysis of the sub population who withdrawn from the study due to airway hyperresponsiveness**

| **Discontinuation due to airway hyperresponsiveness** | **Total n=6** |  |  |
| --- | --- | --- | --- |
| **Spirometry measurements** | **before** | **after** | *p-value* |
| FEV1 Liters | 1.7 (0.61) | 1.7 (0.56) | 0.58 |
| FEV1 % of predicted | 79.8 (28.8) | 84.0 (31.4) | 0.47 |
| FVC Liters | 2.8 (0.6) | 3.0 (0.5) | 0.09 |
| FVC % of predicted | 100.2 (15.4) | 107.3 (14.2) | 0.09 |
| **Etiology** |  |  |  |
| asthma | 2 (33.3) |  |  |
| idiopathic | 2 (33.3) |  |  |
| post-infective | 1 (16.7) |  |  |
| immunodeficiency | 1 (16.7) |  |  |
| **smoking status** | |  |  |
| never | 5 (83.3) |  |  |
| actual | 1 (16.7) |  |  |

**Supplemental 4. Overview of the adverse events and serious adverse events in the total population**

| **Serious adverse events** | **28** |
| --- | --- |
| **Hospital admission** | 28 |
| Protocol defined pulmonary exacerbation | 24 |
| Known cardiac diseases | 2 |
| Near- collapse | 1 |
| Anaphylactic reaction on amoxicillin clavulanate | 1 |
| **Adverse events** | **157** |
| Protocol defined pulmonary exacerbation | 99 |
| Non-protocol defined pulmonary exacerbation | 20 |
| Antibiotics for other reasons | 8 |
| Persistent cough/ dyspnea/ hoarseness | 4 |
| Tinnitus | 3 |
| Headache | 3 |
| Strain muscles | 3 |
| Tiredness | 2 |
| Rectal bleeding/Anemia | 2 |
| Nausea/vomiting | 2 |
| Chest pain | 2 |
| Radiotherapy (treatment malignancy) | 2 |
| Dry mouth | 2 |
| Heart failure | 1 |
| Anaphylactic reaction | 1 |
| Renal dysfunction due to prostate hyperplasia | 1 |
| Renal dysfunction due to diuretics | 1 |
| Renal dysfunction | 1 |
